# Breast Cancer-Derived Microparticles Display Tissue Selectivity in the Transfer of Resistance Proteins to Cells

**DOI:** 10.1371/journal.pone.0061515

**Published:** 2013-04-12

**Authors:** Ritu Jaiswal, Frederick Luk, Penelope V. Dalla, Georges Emile Raymond Grau, Mary Bebawy

**Affiliations:** 1 School of Pharmacy, Graduate School of Health, The University of Technology, Sydney, NSW, Australia; 2 Vascular Immunology Unit, Sydney Medical School and Bosch Institute, The University of Sydney, NSW, Australia; B.C. Cancer Agency, Canada

## Abstract

Microparticles (MPs) play a vital role in cell communication by facilitating the horizontal transfer of cargo between cells. Recently, we described a novel “non-genetic” mechanism for the acquisition of multidrug resistance (MDR) in cancer cells by intercellular transfer of functional P-gp, via MPs. MDR is caused by the overexpression of the efflux transporters P-glycoprotein (P-gp) and Multidrug Resistance-Associated Protein 1 (MRP1). These transporters efflux anticancer drugs from resistant cancer cells and maintain sublethal intracellular drug concentrations. By conducting MP transfer experiments, we show that MPs derived from DX breast cancer cells selectively transfer P-gp to malignant MCF-7 breast cells only, in contrast to VLB_100_ leukaemic cell-derived MPs that transfer P-gp and MRP1 to both malignant and non-malignant cells. The observed transfer selectivity is not the result of membrane restrictions for intercellular exchange, limitations in MP binding to recipient cells or the differential expression of the cytoskeletal protein, Ezrin. CD44 (isoform 10) was found to be selectively present on the breast cancer-derived MPs and not on leukaemic MPs and may contribute to the observed selective transfer of P-gp to malignant breast cells observed. Using the MCF-7 murine tumour xenograft model we demonstrated the stable transfer of P-gp by MPs *in vivo*, which was found to localize to the tumour core as early as 24 hours post MP exposure and to remain stable for at least 2 weeks. These findings demonstrate a remarkable capacity by MPs to disseminate a stable resistant trait in the absence of any selective pressure.

## Introduction

Cell-cell communication is vital for the co-ordination of physiological processes and for the regulation of the organism's phenotype. Cells' communicate via the release of specific molecules i.e. in autocrine signalling, endocrine signalling or across gap-junctions. Cells also communicate by direct cell-cell contact [1] or via supramolecular mechanisms involving cellular membrane blebs/fragments including; membrane vesicles or microparticles (MPs) [2], [3], exosomes [4], [5], apoptotic bodies [6], tunnelling nanotubes [7], [8] and cytoneme or filopodial bridges [9]. Membrane vesicle signaling, although not as complex as that observed with soluble mediators, has been shown to contribute to many distinct processes. These include; microglial-astrocyte interactions [10], coagulation, inflammation [11], [12], cell metabolism [13], [14], HIV-1 [15], cancer progression [16], [17] and drug resistance [2]. The horizontal transfer of mRNAs and miRNAs by tumour derived membrane vesicles have been shown to contribute to conferring numerous malignant traits [3], [18].

Microparticles (MPs) are small (0.1–1 µm in diameter) membrane vesicles formed by the direct budding of the plasma membrane, and which display phosphatidylserine (PS) on their surface [19]. MPs are released from various cell types following a breakdown of membrane asymmetry and cytoskeletal remodeling during the ubiquitous cellular phenomenon of membrane budding [20]. MPs can be distinguished from exosomes by size (exosomes typically ranging 40–100 nm in diameter), as well as by origin [21]. MPs carry a wide range of bioactive material on their surface and serve as carriers of surface antigens, adhesion molecules as well as cellular and nuclear constituents from their originating cells [2], [15], [22]. The presence of cell adhesion molecules on MPs confers a capacity for target cell binding and cell-cell interactions [15].

In 2009, we first described a novel MP mediated “non-genetic” mechanism for the acquisition of multidrug resistance (MDR) in cancer cells, whereby MPs serve as vectors in the intercellular transfer of functional P-gp from MDR donor cells to drug sensitive recipient cells (1). Furthermore, we have since demonstrated that MPs play a more deleterious role in cancer cell biology by incorporating and transferring a variety of nucleic acid species [18] and by “*re-templating*” the transcriptional landscape of recipient cells to ensure the acquisition of deleterious cancer traits [23]. This phenomenon appears further regulated by a process of selective MP packaging and transfer of nucleic acid species to recipient cells [18]. Herein, we expand on these earlier findings to demonstrate that MP transfer of P-gp is a “tissue selective” process dependent on the donor MP type. We demonstrate that the cell adhesion molecule CD44 (isoform 10) is exclusively present on the breast cancer-derived MPs relative to the leukaemic cell-derived MPs. We further propose that the involvement of MP surface molecules and FERM domain proteins could be responsible for the observed transfer selectivity. We also demonstrate that drug resistant MPs can confer the acquired MDR phenotype to recipient cells *in vivo*, resulting in the transfer of P-gp within the tumour core as early as 24 hours post MP exposure. This acquired phenotype was stable for at least 2 weeks in the absence of further MP administration or a selective pressure.

## Materials and Methods

### Cell Lines

In this study, three different types of human malignant cell lines overexpressing P-gp or MRP1 and three human non-malignant primary cells were used. CCRF-CEM (CEM) [24] is a drug-sensitive human acute lymphoblastic leukaemia cell line, of which VLB_100_ [25] (overexpressing P-gp) and E_1000_ [26] (overexpressing MRP1) are its MDR variants. MCF-7 [27] is a drug sensitive human breast adenocarcinoma cell line, of which MCF-7/DX (DX) [28] (overexpressing P-gp) is its MDR variant. MCF-7/P-gp/EGFP is a MCF-7 cell stably transfected with a P-gp enhanced green fluorescent fusion tagged protein [29]. The leukaemic cell lines were a kind gift from Prof Ross Davey (Royal North Shore Hospital, The University of Sydney, NSW, Australia) and the breast cancer cell lines were gifts from Dr Rosanna Supino (Istituto Nazionale per lo Studio e la Cura dei Tumouri, Milan, Italy) and Dr Suzanne M. Cutts (La Trobe University, Victoria, Australia). Both drug sensitive cell lines and the MDR variants have been validated as an appropriate model for the study of P-gp- or MRP1-mediated MDR *in vitro* [2], [30], [31]. All cancer cell lines were cultured in RPMI-1640 (Life Technologies, VIC, Australia) containing 10% FCS (Life Technologies) and maintained under humidified conditions at 37°C in an atmosphere of 5% CO_2_.

Human mammary basal epithelial cells (MBE) obtained from the mammary tissue of a healthy female, were purchased from Zenbio Inc. (NC, USA) and cultured in DMEM/F12 media. The human osteoblasts–femural (HO-f) were cultured in Osteoblast medium and the Human Urothelial cells (HUC) cultured in Urothelial cell medium were obtained from ScienceCell Research Laboratories (CA, USA). These cells were maintained under humidified conditions at 37°C in an atmosphere of 5% CO_2_.

### MP isolation and identification

MPs were isolated from confluent VLB_100_, E_1000_, DX and MCF-7/P-gp/EGFP cells by differential centrifugation, as previously described [2], [23]. The MPs were designated VLBMP, E_1000_MP, DXMP and GFP-MP respectively, for simplicity. Briefly, culture supernatants (cell conditioned media) were collected and centrifuged at 500 g for 5 min to pellet whole cells and debris. The collected supernatant was re-centrifuged at 15,000 g for 1 h at 15°C to pellet the MPs. The final pellet was resuspended in serum free RPMI 1640 media and centrifuged at 2000 g for 1 min to remove debris. The clear MP suspension was further centrifuged at 18,000 g for 30 min at 15°C to pellet MPs. Validation of the isolated MP pellet was performed using flow cytometer (FCM) (BD™ LSR II, BD Biosciences) after FITC-annexin V (Beckman Coulter, NSW, Australia) staining as previously described [2]. Total protein content of MPs was determined using the Quant-iT™ protein assay as per the manufacturer's instructions (Life Technologies).

### MP transfer experiment

In a 96-well U bottom culture plates, 180 µg of VLBMP, E_1000_MP or DXMP were co-cultured with 1×10^5^ MBE, HO-f or HUC cells, for 4 h in a total of 200 µL complete culture medium at 37°C and 5% CO_2_. Unbound MPs were removed by centrifuging at 500 g for 5 min at 25°C after 4 h.

### SDS-PAGE and Western Blotting

Total cellular proteins were separated on 4–12% NuPAGE Bis-Tris gel (Life Technologies) before transferring to PVDF membrane (Pall Australia, VIC, Australia). The membrane was blocked, incubated with anti-P-glycoprotein mAb (clone F4), anti-MRP1 mAb (clone QCRL-1) (Sigma-Aldrich, NSW, Australia), anti-Ezrin mAb (clone 3C12) or anti-CD44 mAb (clone EPR1013Y) (Abcam, MA, USA). Anti-α-tubulin (clone DM1A) or anti-β-actin (clone AC-74) (Sigma-Aldrich) were used as the internal controls, followed by horseradish peroxidase-conjugated secondary antibody and subjected to enhanced chemiluminescence (Roche, VIC, Australia).

### Confocal microscopy

Confocal microscopic experiments were performed on co-cultures to complement the Western blot studies. Microparticles (GFP-MPs) were isolated from MCF-7/P-gp/EGFP breast cancer cells. Malignant drug sensitive MCF-7 cells or the non-malignant osteoblasts (HO-f cells) were co-cultured with equal amounts of the GFP-MPs for 4 hours on coverslips. Following co-culture, unbound MPs were removed by rinsing twice with PBS for 10 mins. The cells were fixed with 2% paraformaldehyde (Sigma-Aldrich) in PBS for 30 mins followed by two rinses with PBS. The fixation was quenched with 100 mM glycine (Research Organics Inc. OH, USA) for 5 mins. The cells were labelled with 200 nM of the cell membrane dye PKH26 (Sigma-Aldrich) for 1 min according to the manufacturer's recommendation followed by two rinses with PBS. The nucleus was stained with 300 nM DAPI (Sigma-Aldrich) for 5 mins. Following two rinses with PBS, the coverslips were mounted on slides with Prolong Gold Antifade Reagent (Life Technologies). Images were acquired utilising confocal laser scanning microscopy on a Nikon A1 microscope (Nikon, Tokyo, Japan).

### Flow cytometry

For the detection of cell surface CD44 in the breast cancer cells, MCF-7 and DX cells were harvested either by scraping gently from the tissue culture flask using a cell scraper or by trypsinising. The leukaemic cells were harvested by centrifuging the cell suspension at 500 g for 5 mins to obtain the cell pellet. 5×10^5^ cells were labelled with 30 µL anti-CD44 (1∶30) (mAb, clone EPR1013Y) and incubated for 30 mins at room temperature in the dark. Samples were washed three times in ice cold DPBS (Sigma-Aldrich) by centrifuging at 500 g for 5 min at 25°C followed by incubation with Alexa Fluor 647-conjugated goat anti-rabbit IgG (Life Technologies) (1∶400 dilution) for 30 mins at 4°C. The cells were washed three times in DPBS as described above and resuspended in 200 µL ice cold 3% BSA in DPBS and analysed by flow cytometry (BD™ LSR II, BD Biosciences).

### Binding of MPs to Non-malignant cells

The extent of fluorescence transfer of PKH26 labelled DXMPs to non-malignant cells following co-culture was performed as previously described [2]. Briefly, after MP purification, DXMPs were labelled with PKH26 red dye (Sigma-Aldrich) according to the manufacturer's recommendation and designated PKH26-DXMP. 1×10^5^ non-malignant breast epithelial cells and osteoblasts were co-cultured with PKH26-DXMP for 4 h at 37°C in complete RPMI-1640 media. Cells were washed with DPBS and centrifuged at 500 g for 5 min to remove unbound MPs. The cells were re-suspended in 200 µl DPBS and analysed using flow cytometry (BD™ LSR II, BD Biosciences).

### *In vivo* experiments

The use of animals in this study was approved by the UTS Animal Care and Ethics Committee (ACEC) at the University of Technology, Sydney (Permit No: 2011-321A) and the experiments were conducted in accordance with the UTS (ACEC) approved protocol. 30 BALB/c athymic nude female mice (4–6 weeks old), weighing 15–20 g were obtained from the Animal Resources Centre (WA, Australia). The animals were kept in groups of five under sterile conditions in filter top cages and were provided with sterilized food and water *ad libitum* throughout the experiment. The mice were allowed to acclimatize in standard conditions (under a 12 hr light/dark cycle) for 8 days prior to any experimental procedures.

#### (i) Tumour induction

MCF-7 and DX tumour xenografts were established as described by Ullmann and colleagues, 1991 [32]. The DX xenograft model has been validated as remaining resistant and retaining the characteristics of MDR as displayed by the cells in culture [32]. The MCF-7 and DX cells require oestrogen for tumour growth *in vivo* [33] and hence, a 90-day slow release 17-β-estradiol pellets (1.7 mg/pellet) (Innovative Research of America, FL, USA) were implanted subcutaneously (s.c.) into the dorsum of each mice under general anaesthesia (2% isoflurane and 0.5–1% oxygen) one week prior to tumour cell injection. Transplantable MCF-7 or DX cells were harvested and suspended in RPMI supplemented with 10% FCS. All mice received s.c. injections of 10^7^ cells/100 µL of inoculation in between the shoulder blades.

When the s.c. tumour reached a palpable size after ∼10 days, the mice were randomised into 2 groups (15 mice each) including MCF-7 tumour bearing mice (10) and DX tumour bearing mice (5) in each group. MPs were isolated from *in vitro* cultures of the DX cells and designated as DXMP. Five MCF-7 tumour bearing mice from each group received injections of 100 µg MP/200 µL of RPMI supplemented with 10% FCS subcutaneously surrounding the tumour periphery. All the other mice (MCF-7 and DX tumour bearing mice) from both groups served as controls and received 200 µL of saline injections. The animals' weight and tumour volume was measured routinely during the course of the experiment. Tumour volume (V) was measured in two perpendicular diameters (A and B) using digital callipers (Dick Smith, NSW, Australia) and calculated based on the formula: **V = π/6 (A+B/2)^3^.**

The animals were further divided into 2 groups; fifteen animals for 24 h and fifteen for 14 days post injection monitoring. Following 24 h post injection, all the mice in the respective group were euthanized by CO_2_ inhalation. Tumours, lungs, livers and kidneys were excised and preserved in 10% neutral buffered formalin solution (Sigma-Aldrich) and embedded in paraffin. Both haematoxylin and eosin (H&E) staining and immunohistochemical detection were performed on tissue sections.

#### (ii) Immunohistochemistry

DakoCytomation EnVision® + Dual Link System-HRP (DAB+) kit (Dako, VIC, Australia) was used for immunohistochemistry staining. 5 µm sections from formalin-fixed and paraffin-embedded tissues were deparaffinised, rehydrated and treated for 20 mins at 95°C in citrate antigen retrieval buffer (pH 6.0) in a water bath. After cooling to room temperature, slides were blocked with the Dual Endogenous Enzyme block (from Dako kit) for 10 mins. The slides were rinsed with distilled water and kept in PBS-T (0.05% Tween 20 in PBS) for 5 mins. Sections were incubated overnight at 4°C with mouse monoclonal anti-P-glycoprotein (1∶100) clone F4 (Sigma-Aldrich) or mouse isotype IgG1 (1∶100) (Cell Signaling, MA, USA) diluted in 1% bovine serum albumin (Sigma-Aldrich). Sections were washed in PBS-T three times for 5 mins each and subsequently incubated with labelled Polymer-HRP (from Dako kit) for 1 h at room temperature. Following four washes with PBS, substrate-chromogen solution (DAB+) was applied for 15 mins. The sections were counterstained with filtered Harris's haematoxylin (Thermo Fisher Scientific, NSW, Australia) for 30 sec, dehydrated by washing in a series of ethanol at increasing concentrations, and mounted with a coverslip with Eukitt® quick-hardening mounting medium (Sigma-Aldrich). The sections were visualized with the Olympus BX51 microscope and images captured using the Olympus DP 70 (Olympus, Japan) camera.

### Statistical analysis

GraphPad Prism software was used to plot the data and a one-way analysis of variance (ANOVA) was used for comparison and statistical analysis between the sample populations. *P* values less than 0.05 (*p*<0.05) were accepted as statistically significant.

For the animal experiments, the study was carried out on 30 mice with equal numbers in each of the 3 treatment groups in order to be statistically significant. The comparison of groups was based on *in vitro* P-gp expression experiments using the sample size calculator of the Minitab software version 15.1. The power, with a two-sided significance level of 5%, to detect our smallest observed increase in P-gp expression following MP transfer in our *in vitro* pilot data (13%) with 10 mice per group was >80%.

## Results

### Breast Cancer-derived MPs Selectively Transfer P-gp to Malignant Breast Cells

**Figure 1:**
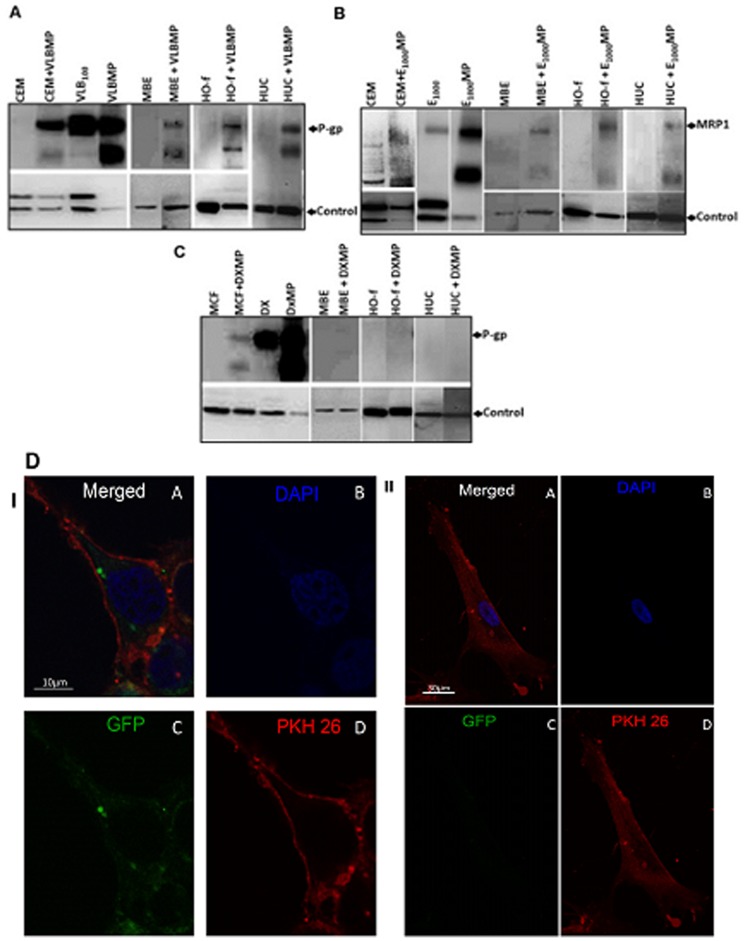
Selective transfer of P-gp to MCF-7 breast cancer cells by DXMPs by Western Blot analysis. 20 µg total cell lysates of non-malignant cells (breast epithelial cells (MBE), osteoblasts (HO-f) and urothelial (HUC) cells) and their co-cultures; and drug sensitive cancer cells (leukaemia, CEM and breast cancer, MCF-7) and their co-cultures with leukaemic cell-derived (**A**) VLBMP and (**B**) E_1000_MP overexpressing P-gp and MRP1, respectively, and breast cancer-derived (**C**) DXMP overexpressing P-gp, were analyzed by Western blot analysis. (**A**) P-gp and (**B**) MRP1 were readily detected in the drug resistant leukaemic donor cells, their MPs and also in both the recipient malignant and non-malignant cells after MP transfer using the anti-P-gp mAb, F4 and anti-MRP1 mAb, QCRL-1, respectively. (**C**) P-gp was also detected in the drug resistant breast cancer donor cell, its MPs (DXMP), recipient malignant drug sensitive cells but not in the recipient non-malignant cells after MP transfer. α-tubulin or β-actin were used as internal loading controls. (**D**) **Selective transfer of P-gp to MCF-7 breast cancer cells by malignant breast cancer-derived MPs by Confocal microscopy.** Malignant breast cells MCF-7 (**I**) or non-malignant osteoblasts, HO-f (**II**) cells were stained with fluorescent membrane probes PKH26 and the nucleus was stained with DAPI after 4 hours co-culture with GFP-MPs. (**I**) P-gp was detected in the breast MCF-7 cells only and not in (**II**) the osteoblasts. Panel A shows all channels captured, Panel B shows the DAPI in the 405 nm channel, Panel C shows the transferred GFP (or lack of) in the 488 nm channel, and Panel D shows the PKH26 in the 561 nm channel. Images were acquired using the Nikon A1 confocal microscope. Scale bar as indicated. Both Western blot and confocal microscopy experiments were repeated at least 2–3 times with similar results. Data are representative of a typical experiment.

To establish whether the drug resistant cancer-derived MPs transferred the drug transporter proteins to the non-malignant cells, we conducted a Western blot analysis probing for acquisition of the transporters, P-gp and MRP1. P-gp was identified at 170 kDa and MRP1 at 190 kDa. P-gp was overexpressed in both the leukaemic and breast cancer resistant cells as well as in their shed MPs (Figure 1A and C). Likewise MRP1 was overexpressed in the leukaemic resistant cell and its shed MPs (Figure 1B). Both P-gp and MRP1 were also detected in all the drug sensitive cancer cells following MP co-culture with MPs isolated from drug resistant cells (Figure 1), consistent with our previous findings [2], [23]. Likewise P-gp expression was detected in all non-malignant cells following co-culture with resistant leukaemic cell-derived MPs (VLBMP) (Figure 1A). Similarly, MRP1 was detected in all non-malignant cells following resistant leukaemic cell-derived MP (E_1000_MP) co-culture (Figure 1B). Interestingly, P-gp was not detected in any non-malignant cells, following co-culture with DXMPs (Figure 1C). Rather, we observed a selective transfer of P-gp by resistant breast cancer-derived DXMPs to malignant breast, MCF-7 cells only (Figure 1C).

In addition, consistent with the Western blot studies, we showed by confocal microscopy that the malignant breast cancer-derived GFP-MPs bind and transfer P-gp to malignant MCF-7 cells only (Figure 1D: IA) and not to the non-malignant osteoblasts (Figure 1D: IIA).

### Transfer Selectivity is Not a Result of Limitations in MP Binding to Recipient Cells

**Figure 2:**
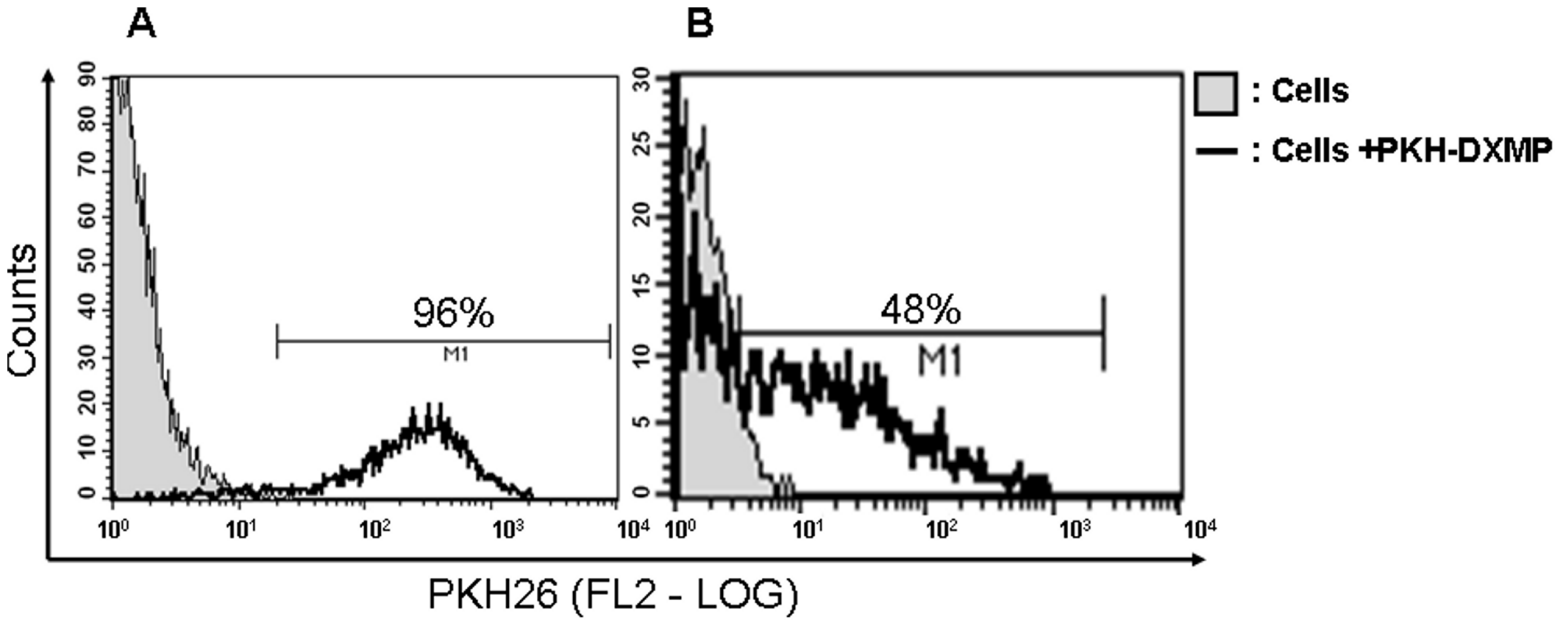
Detection of PKH-26 labeled DxMP binding to normal cells by flow cytometry (FCM). After purification and staining, the co-incubation of PKH-26 DXMP labelled DXMPs (**A**) 96% of the breast epithelial (MBE) cells (black histogram) and (**B**) 48% of the osteoblasts (HO-f) showed PKH26 fluorescence (black histogram) with respect to the cells with no MP incubation (gray histograms). The experiment was repeated at least 2–3 times with similar results. Data are representative of a typical experiment.

To establish whether the lack of DXMP transfer of P-gp to non-malignant cells was the result of limitations in MP binding to the recipient cells, PKH26-labelled DXMP were incubated with either the human breast epithelial cells (MBE) or the human osteoblasts (HO-f). Following a 4 h co-incubation, flow cytometric analysis of the resultant recipient breast epithelial cells displayed a 96% (Figure 2A) and the osteoblasts a 48% (Figure 2B) increase in PKH26 labelling compared to the cells alone, consistent with the binding of PKH26-DXMP to the surface of these cells.

### Ezrin is Selectively Packaged in Cancer-derived MPs

**Figure 3:**
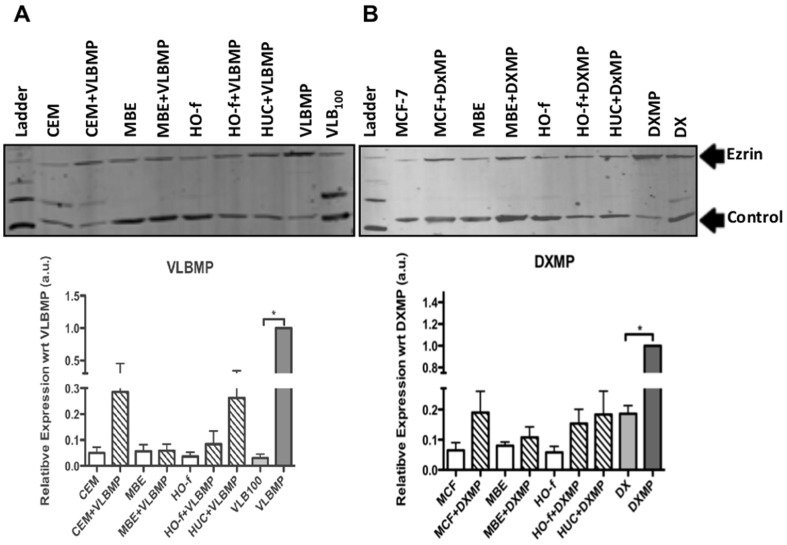
Breast cancer-derived MPs transfer Ezrin to recipient cells. 17.5 µg total cell lysates of breast epithelial cells, osteoblasts and urothelial cells and their co-cultures with (**A**) leukaemic cell-derived MPs namely VLBMP and (**B**) Breast cancer-derived MPs namely DXMP both overexpressing P-gp were analyzed by Western blot analysis followed by densitometric quantification after normalization to total protein loaded to the gel. (**A**) The presence of Ezrin in the leukaemic cell-derived VLBMP and in the recipient cells after MP transfer was readily detected using the anti-Ezrin mAb, 3C12. (**B**) Ezrin was also detected in the breast cancer DXMP and in all the recipient cells after MP transfer. β-actin was used as an internal loading control. The Western blot experiments were repeated at least three times with similar results. Data are representative of a typical experiment. Densitometric data represent the mean ± SEM of 3 independent experiments * *p*<0.05.

Since Ezrin plays a role in P-gp membrane insertion through a cytoskeletal association [34], we sought to examine whether Ezrin levels correlated with the transfer selectivity observed. Ezrin was present in both leukaemic and breast cancer-derived MPs, where it was selectively packaged relative to the donor cells (Figure 3). We observed no significant differential expression of Ezrin in the recipient cells beyond their endogenous levels present, before or after co-culture (Figure 3) and hence transfer selectivity does not appear related to differential levels of Ezrin.

### The Cell Adhesion Molecule CD44 is Selectively Present on Breast Cancer-derived MPs

**Figure 4:**
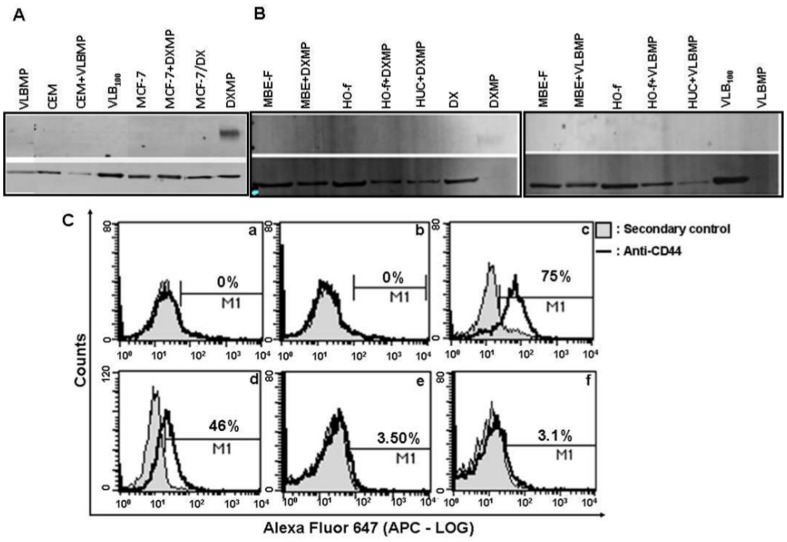
Breast cancer MPs carry but do not transfer CD44 to recipient cells. 20 µg total cell lysates of (**A**) drug sensitive cancer cells (leukaemia, CEM and breast cancer, MCF-7) and their co-cultures; (**C**) non-malignant cells (breast epithelial cells (MBE), osteoblasts (HO-f) and urothelial (HUC) cells) and their co-cultures; with leukaemic cell-derived VLBMP and breast cancer-derived DXMP both overexpressing P-gp were analyzed by Western blot analysis. (**A**) CD44 was only detected in the breast cancer DXMP but not in the malignant MCF-7 cell (**A**), or in any of the non-malignant cells (**B**) following DXMP co-culture. (**A**) CD44 was not detected in the leukaemic cell-derived VLBMP, the malignant cells or (**B**) the non-malignant cells follwoing VLBMP co-culture using the anti-CD44 mAb, clone EPR1013Y. β-actin or α-tubulin or were used as an internal loading control. The Western Blot experiments were repeated at least three times with similar results. (**C**) **Detection of CD44 on breast cancer and leukaemic cells by flow cytometry**. MCF-7 (**a**–**b**) and DX (**c**–**d**) cells were harvested from culture flasks either by scraping (a and c) or by trypsinising (b and d). Leukaemic CEM (**e**) and VLB_100_ (**f**) cells were harvested by centrifuging the cell suspension. Cells were surface labelled with anti-CD44 (1∶30) (mAb, clone EPR1013Y) followed by Alexa Fluor 647-conjugated goat anti-rabbit IgG (1∶400). No CD44 was detected on the MCF-7 cells harvested without (**a**) or with trypsin (**b**). 75% DX cells harvested without (**c**) or 46% DX cells harvested with trypsin (**d**), were detected positive for CD44 with respect to the secondary control by flow cytomtery. Almost equal percentage (3.1–3.5%) of CEM (**e**) and VLB_100_ (**f**) cells were detected positive for CD44 with respect to the secondary control. Data are representative of a typical experiment.

Since CD44 has been previously shown to associate with P-gp and the cytoskeleton, we sought to assess whether CD44 was present in DXMP, whether it was differentially expressed with respect to VLBMPs and as such could account for the observed transfer selectivity. CD44 (isoform 10) was found to be selectively packaged in the breast cancer-derived DXMP relative to its donor cells, where surprisingly no CD44 was detected by Western blot (Figure 4A). However, CD44 was detected in the donor DX cells by flow cytometry, where 75% of the cell population harvested without trypsinising stained positive for CD44 whereas only 46% stained positive for CD44 following cell harvest using trypsin (Figure 4C [a–d]). In contrast, insignificant levels (3–3.5%) of CD44 was detected in the leukaemic cells (Figure 4C [e–f]). Likewise, CD44 was not present in the leukaemic cell-derived VLBMP (Figure 4A) or any of the malignant (Figure 4A) or non-malignant cells (Figure 4B) following both DXMP and VLBMP co-cultures (Figure 4). This result demonstrates that CD44 (isoform 10) is selectively present in DXMPs however it is not transferred to recipient cells.

### MP Transfer of P-gp to Breast Cancer Cells Localizes to the Tumour Core and is Stable for at Least Two Weeks *In Vivo*

**Figure 5:**
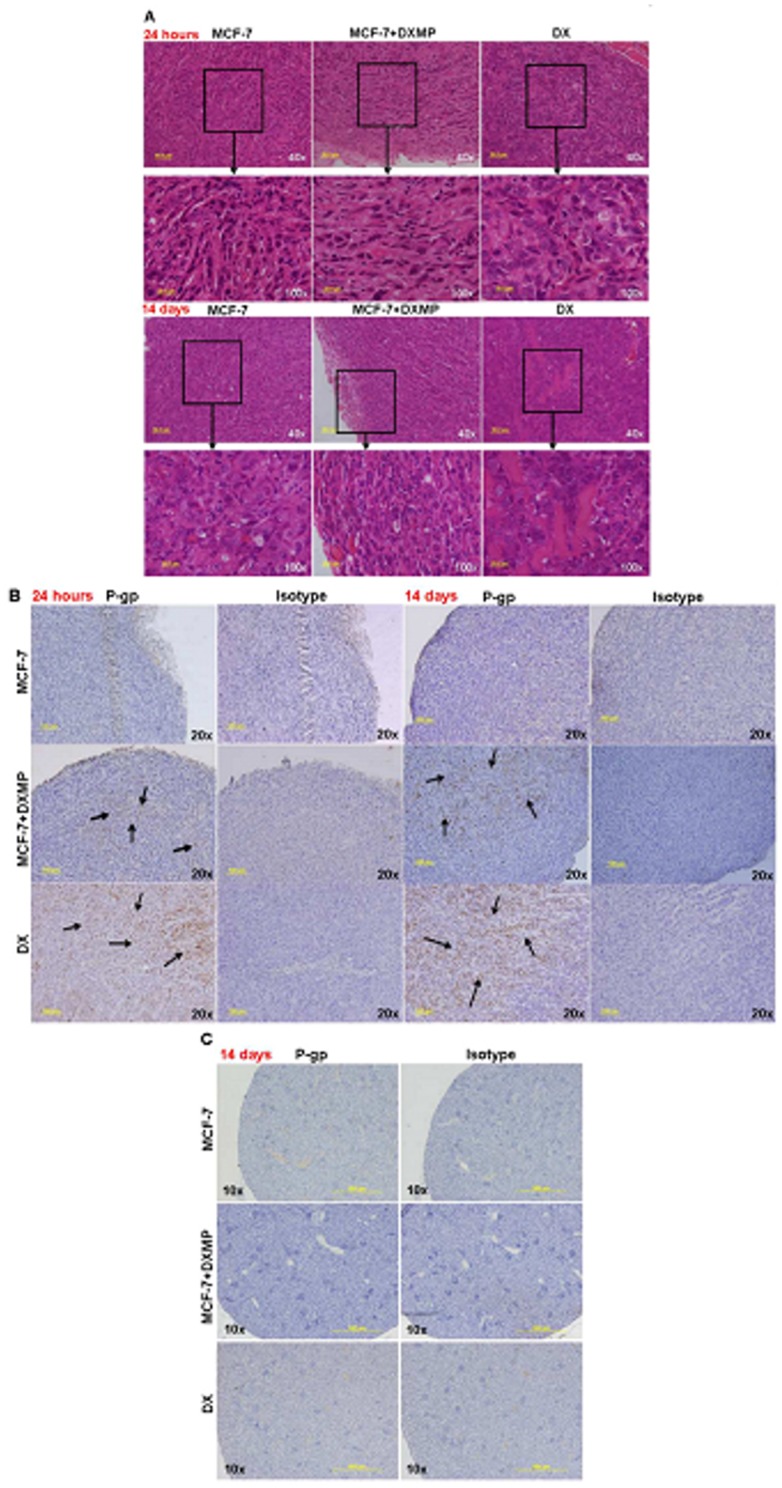
P-gp localised and retained in tumour core, two weeks post MP administration. At 10 days after tumour cell implantation, tumours derived from female athymic mice bearing MCF-7 (negative control) and DX (positive control) or MCF-7 tumours with DXMP exposure (MCF-7+DXMP) were resected and fixed after 24 h and 14 days DXMP exposure. Five independent tumours (each from a different mouse) were tested in each mouse group. (**A**) Excised and sectioned tumour slides were subjected to haematoxylin and eosin (H&E) staining showing the presence of breast epithelial cells and blood vessels in the tumour sections at both 24 h and 14 days. Slides were examined at 40× followed by 100× magnification. (**B**) Tumour and (**C**) kidney specimens were subjected to immunohistochemical (IHC) staining with antibodies specific to P-glycoprotein (anti-P-gp mAb, F4) and its Ig G isotype control. (**B**) P-gp (indicated in black arrows) was found in MCF-7 tumours, 2 weeks post DXMP exposure. P-gp was observed in MCF-7 tumours (-ve control) and DX tumours (+ve control). (**C**) No P-gp was observed in kidney 14 days after DXMP administration. Slides were examined at 10× or 20× magnification. Images are of a representative animal.

To ascertain MP-mediated acquisition of P-gp in the *in vivo* state, we examined P-gp transfer by DXMPs using the MCF-7 tumour xenograft model. Female athymic nude mice were used for the drug sensitive (MCF-7) and the drug resistant (DX) tumour xenografts. Breast epithelial cells were confirmed in all tumour samples by histopathological examination (Figure 5A). P-gp was acquired following a single subcutaneous injection of DXMPs surrounding the tumour periphery. P-gp localized to the tumour core of recipient breast MCF-7 tumours at both 24 h and 14 days post MP exposure (Figure 5B). P-gp expression was stable for at least 2 weeks following a single dose of DXMPs (Figure 5B) in the absence MP re-administration or any selective pressure.

DXMP transfer of P-gp was not observed at the level of kidney (Figure 5C) or lung (data not shown).

## Discussion

In this study we show that MPs display "tissue selectivity" in transferring cancer traits to recipient cells. Specifically, MPs shed by multidrug resistant DX breast cancer cells, selectively transfer resistance proteins to malignant MCF-7 breast cells (Figure 1C, 1D: I). Contrary to this however, leukaemic cell-derived MPs (VLBMPs), effectively transfer P-gp (Figure 1A) and MRP1 (Figure 1B) to non-malignant and malignant recipient cells. The observed selectivity was not the result of limitations in MP binding to recipient cells (Figure 2). Nor was it the result of limitations of recipient cell plasma membranes for intercellular exchange, given the non-selective crosstalk observed by the leukaemic cell-derived MPs. The basis of the observed transfer selectivity thus appears not to reside at the recipient cell membrane but rather at the level of MPs. As P-gp resides both inside and outside membrane rafts, membrane ultrastructures at the level of the MPs appears unlikely to govern the observed transfer selectivity [35]. On this basis we further propose that the specificity displayed by the cancer derived DXMPs likely resides at the protein level.

Ezrin, through cytoskeletal association, is known to play a role in P-gp membrane localization. The actin-P-gp interaction was also shown to be required for the endosomal trafficking of P-gp to the plasma membrane [29], [34]. Disruption of the ERM–P-gp association impairs P-gp function and results in a cellular redistribution of P-gp [34], supporting an essential role for the ERM proteins in the plasma membrane localization of P-gp. Given the role of Ezrin in P-gp membrane insertion, we examined whether Ezrin levels correlated with transfer selectivity. Ezrin was shown to be selectively packaged in both leukaemic and breast cancer-derived MPs relative to their donor cells (Figure 3). However, we observed no difference in levels across both malignant and non-malignant cells pre and post MP exposure. Our results, establish that the transfer selectivity observed by DXMPs does not directly reside at the level of Ezrin expression but may reside with other cytoskeletal associated proteins or their effectors.

**Figure 6:**
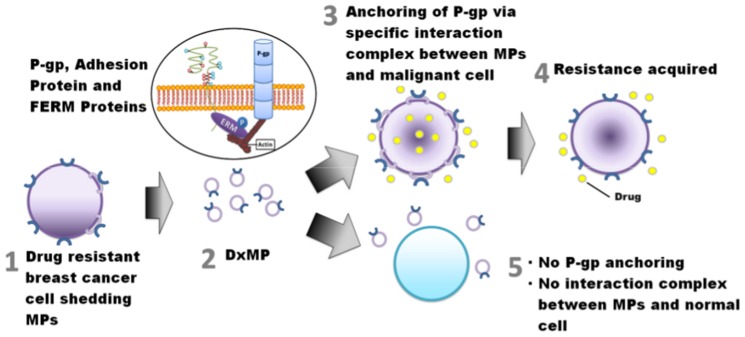
Hypothesis Overview. An interaction complex involving discrete MP surface molecules and FERM domain proteins (including Ezrin) serve to selectively target and anchor P-gp to breast cancer cells upon MP binding. This leads to the selectivity in dissemination and acquisition of MDR trait in cells.

Another cell adhesion molecule, CD44, which interacts with P-gp via FERM domain binding proteins, was also assessed in understanding the basis of the transfer selectivity observed for breast cancer-derived DXMPs. We observed that CD44 (isoform 10) was selectively packaged by the DXMPs relative to the donor cells where its presence was undetected by Western blot (Figure 4A). When assessed by flow cytometry however, the donor DX cells were positive for CD44 (Figure 4C, c–d). This implies that the CD44 protein in the donor cells may be prone to degradation and/or have altered conformation due to trypsin treatment and/or lysis. Hence, the cell surface CD44 protein is only detected by flow cytometric analysis on the live cells without lysis or trypsinisation (Figure 4C [a–d]). In contrast, we failed to detect significant levels of the CD44 isoform 10 in the leukaemic cells (not treated by trypsin) by flow cytometry (Figure 4C [e–f]). Likewise, no CD44 was detected in the drug resistant cell-derived VLBMP and in any of the recipient cells following both DXMP and VLBMP co-cultures (Figure 4B) by Western blot. This observation, in addition to CD44's exclusivity of presence in the breast cancer-derived MPs is indeed surprising and requires further examination. These results also demonstrate that although CD44 is present in DXMPs, it is not transferred. Rather, its presence in MPs may be required to stabilise P-gp within the MP membrane whilst *en route* to recipient cells and may have an important role at the MP level for the selective transfer of P-gp to malignant breast cells whilst it is not transferred itself. Given the cytoskeleton plays an important role in MP biogenesis and the membrane localisation of P-gp via the FERM domain proteins, we hypothesise that an interaction complex involving discrete MP surface molecules and FERM domain proteins (like Ezrin) may serve to selectively target and anchor P-gp to breast cancer cells upon MP binding (Figure 6).

In addition to cell adhesion molecules, there are a diversity of other molecules such as cellular proteins, growth factors, second messengers and other nucleic acids species (RNA and miRNAs) that have been implicated in intercellular communication via membrane vesicles [3], [18]. Indeed we have shown the transfer of not only functional P-gp [2] but also RNAs and miRNAs [18], [23] via MPs leading to the re-templating of the recipient cells to ensure acquisition of the donor cell MDR trait [18], [23]. In addition to the involvement of these entities in the transfer selectivity observed by breast cancer-derived MPs, it may be worth examining the role of other mechanisms of intercellular communication in the transfer selectivity as well as dissemination selectivity of MDR traits in cancer.

Finally, the transfer of P-gp by DXMPs in the *in vivo* state was examined using a breast cancer (MCF-7) tumour xenograft model in athymic nude mice. Remarkably we observed that P-gp was acquired following a single subcutaneous injection of DXMP surrounding the tumour mass and was localized to the tumour core of the recipient breast MCF-7 tumours (Figure 5B) within 24 hours of MP administration. P-gp expression was stable for at least 2 weeks following a single dose of DXMPs (Figure 5B), in the absence of any selective pressure such as drugs or MP re-exposure. DXMP transfer of P-gp was not observed in the non-malignant organs such as kidney (Figure 5C) and the lung (data not shown). Endogenous P-gp was detected in the liver, however the levels were no different from untreated controls (data not shown). The absence of P-gp detection in these organs could possibly be attributed to the subcutaneous injection of MPs, which were not injected in the immediate vicinity of these organs. Hence, future studies will involve examining the effects on other organs, following intravenous administration of MPs. Our results validate for the first time the significance of MP mediated transfer of P-gp *in vivo* and more importantly demonstrate a prolonged retention of transferred P-gp following a single dose, even in the absence of a selective pressure. It is currently unknown whether for a tumour to develop the MDR phenotype, it is necessary only for a few cells to acquire the phenotype or the majority of the cells in the tumour to develop the resistance independently. It will also be interesting to explore in future whether a single cell has the potential to pass onto the acquired trait to its neighbouring cells or to its daughter cells.

In conclusion, we demonstrate for the first time the acquisition and the stability of MDR mediated by MPs in cancer cells *in vivo.* The stable resistance acquired by MP-mediated P-gp transfer *in vivo* has the potential to provide the drug sensitive cells a quick survival advantage and a barrier to xenobiotic exposure, making cancer treatment even more challenging. In addition, we also report that MP-mediated transfer of P-gp is a "tissue selective" process dependent on the donor MP type.
